# Supplementary material for: Transfer learning enables discovery of sub-micromolar antibacterials for ESKAPE pathogens from ultra-large chemical spaces
Source: Chem Sci. 2025 Sep 16;16(45):21518–33. doi: 10.1039/d5sc03055b (PMC12523573; doi:10.1039/d5sc03055b)
Supplement: SC-016-D5SC03055B-s001 [file SC-016-D5SC03055B-s001.pdf]

Supplementary Information (SI) for Chemical Science.
This journal is © The Royal Society of Chemistry 2025

Supplementary Information

Supplementary Information S1. Inhibition loss function

We developed a custom loss function, called the inhibition loss l_i(IL), which takes as input values in the range [0,1]. The IL was designed to address the limitations of standard regression or classification losses.

The IL is based on a hard binary classification loss (denoted as l_h), which imposes a unit penalty for each false positive or false negative. The hard loss l_h can be written as:

$$l_h(y,\hat{y}) := I(\hat{y})\,[1 - I(y)] + I(y)\,[1 - I(\hat{y})]$$

where the first term penalizes false positives and the second term penalizes false negatives. Here, $y \in \{0,1\}$ are the true values, $\hat{y} \in \{0,1\}$ are the predicted values, and $I(\cdot)$ is the indicator function:

$$I(x) = 0 \text{ if } x \leq 0.5$$

$$I(x) = 1 \text{ if } x > 0.5$$

This hard loss l_h can be approximated by a soft loss l_s by replacing the step function with a sigmoid squashing function $\sigma : R \rightarrow [0,1]$, $\sigma(x) = \frac{1}{1 + \exp(-x)}$

Thus, the soft loss s is:

$$l_s(y,\hat{y}) := \sigma(\hat{y})\,[1 - \sigma(y)] + \sigma(y)\,[1 - \sigma(\hat{y})]$$

While the sigmoid function σ has its midpoint at $x = 0, g(0) = 0.5$, this is not always appropriate for all classification tasks. In particular, in our work, we adopted a binarization threshold of 0.8 for antibacterial activity, as suggested by Stokes et al.[12] To achieve this, we modified the squashing function to ensure that its midpoint occurs at $x = 0.8$. This modification is represented by:

$$g(x) := \sigma\left(c_1\left(e^{c_2(x - 0.8)} - 1\right)\right)$$

where c_1 controls the range of the squashing function (ensuring $g(0) \approx 0$ and $g(1) \approx 1$), and c_2 adjusts the steepness of the slope at $g(0) = 0.5$. In our experiments, we set $c_1 = 4$ and $c_2 = 3.28$.

The final inhibition loss (l_i) is defined as:

$$l_i(y,\hat{y}) := c_+ g(\hat{y}) [1 - g(y)] + c_- g(y) [1 - g(\hat{y})]$$

where c_+ and c_- are coefficients that scale the penalties for false positives and false negatives, respectively. These coefficients were treated as hyperparameters and optimized during training, with values $c_+ \in [0,1]$, $c_- \in [0,1]$, and $c_+ + c_- = 1$.

Supplementary Figures

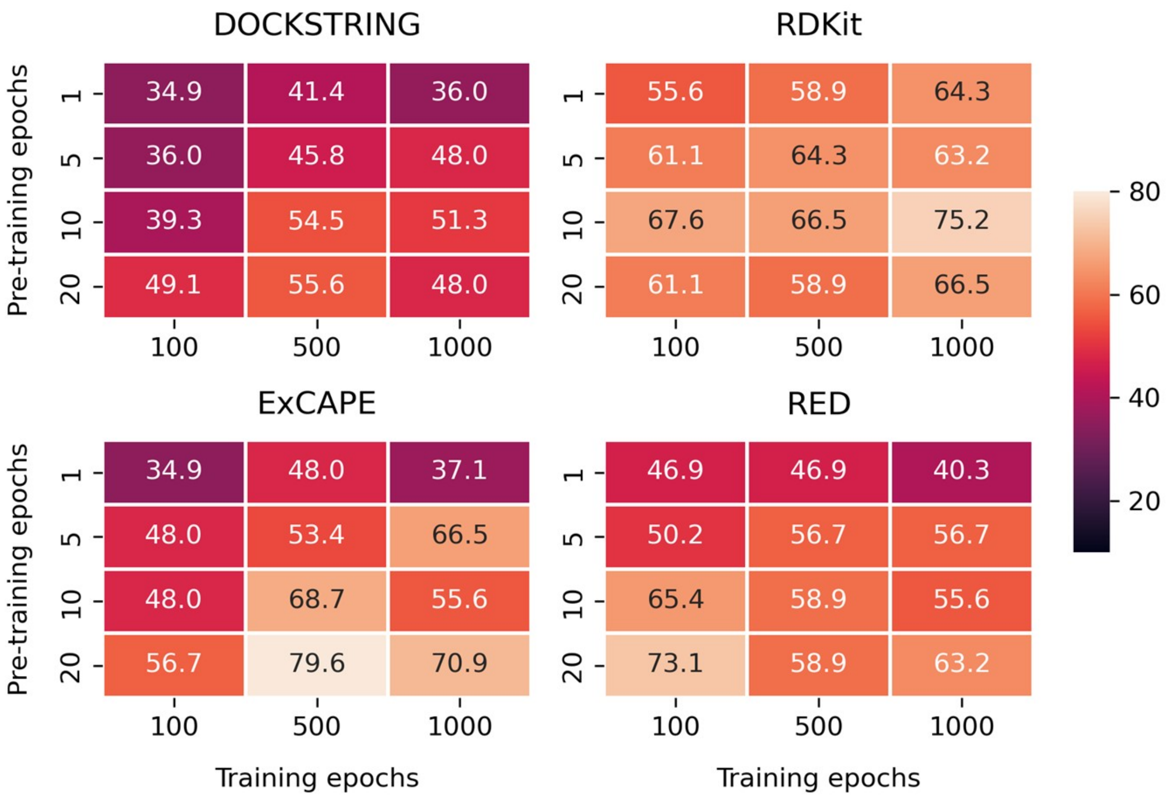

Supplementary Figure S1. Enrichment Factors obtained in the test set from the various models in the process of optimizing the hyperparameters related to the transfer-learning protocol: the choice of a pre-training dataset, the number of pre-training epochs, and the number of fine-tuning epochs.
